# ArmAssist Robotic System versus Matched Conventional Therapy for Poststroke Upper Limb Rehabilitation: A Randomized Clinical Trial

**DOI:** 10.1155/2017/7659893

**Published:** 2017-01-31

**Authors:** Tijana J. Dimkić Tomić, Andrej M. Savić, Aleksandra S. Vidaković, Sindi Z. Rodić, Milica S. Isaković, Cristina Rodríguez-de-Pablo, Thierry Keller, Ljubica M. Konstantinović

**Affiliations:** ^1^Clinic for Rehabilitation “Dr. Miroslav Zotović”, Faculty of Medicine, University Belgrade, Sokobanjska 13, 11000 Belgrade, Serbia; ^2^Tecnalia Serbia Ltd., Vladetina 13 and Signals and Systems Department, School of Electrical Engineering, University of Belgrade, Bulevar kralja Aleksandra 73, 11200 Belgrade, Serbia; ^3^Neurorehabilitation Area at the Health Division of TECNALIA, San Sebastian, Spain

## Abstract

The ArmAssist is a simple low-cost robotic system for upper limb motor training that combines known benefits of repetitive task-oriented training, greater intensity of practice, and less dependence on therapist assistance. The aim of this preliminary study was to compare the efficacy of ArmAssist (AA) robotic training against matched conventional arm training in subacute stroke subjects with moderate-to-severe upper limb impairment. Twenty-six subjects were enrolled within 3 months of stroke and randomly assigned to the AA group or Control group (*n* = 13 each). Both groups were trained 5 days per week for 3 weeks. The primary outcome measure was Fugl-Meyer Assessment-Upper Extremity (FMA-UE) motor score, and the secondary outcomes were Wolf Motor Function Test-Functional Ability Scale (WMFT-FAS) and Barthel index (BI). The AA group, in comparison to the Control group, showed significantly greater increases in FMA-UE score (18.0 ± 9.4 versus 7.5 ± 5.5, *p* = 0.002) and WMFT-FAS score (14.1 ± 7.9 versus 6.7 ± 7.8, *p* = 0.025) after 3 weeks of treatment, whereas the increase in BI was not significant (21.2 ± 24.8 versus 13.1 ± 10.7, *p* = 0.292). There were no adverse events. We conclude that arm training using the AA robotic device is safe and able to reduce motor deficits more effectively than matched conventional arm training in subacute phase of stroke. The study has been registered at the ClinicalTrials.gov, ID: NCT02729649.

## 1. Introduction

Stroke is the leading cause of long-term disability in the industrialized world [1]. Up to 85% of stroke survivors experience arm weakness and only 20%–56% regain complete motor function at 3 months [2, 3]. Not surprisingly, the degree of recovery in motor function has the greatest impact on subjective well-being at 1 year after stroke [4].

The main principle of contemporary rehabilitation is a task-specific practice with a large number of repetitions, which is a potent stimulus for promoting motor learning [5, 6]. Studies on the dose-response relationship have shown that more therapy is associated with better motor recovery. Nevertheless, conventional treatments are still not delivered as intensively or frequently as necessary because of the cost, repetitive strain injuries in patients, and limited availability of therapists [5].

Various robot-assisted systems have been developed for enhancing arm motor recovery, including unilateral, bilateral, proximal arm and distal arm training devices. Robots encourage high repetition of movements with minimal supervision in a highly motivating environment. Robotic devices should provide therapy relevant to users and conform to the principles of conventional therapy. They should be easy to set up, appeal to users, require minimal supervision from therapists, and ideally be able to assess motor performance and therapy outcomes [7, 8]. A recent review concluded that robotic systems may improve motor control of upper limbs after stroke but not necessarily increase functional abilities [9]. Despite potential for improving upper limb functions after stroke, the quality of evidence in support of robot devices remains low [10].

A few simple and low-cost robotic devices have been evaluated in stroke patients with moderate arm weakness. Improvements in kinematic and clinical outcomes were reported in moderately disabled chronic stroke patients who participated in a telerehabilitation program using a simple robotic device [11]. The study also found high level of interest, motivation, and enjoyment among patients with minimal supervision from the therapist [11].

**Figure 1:**
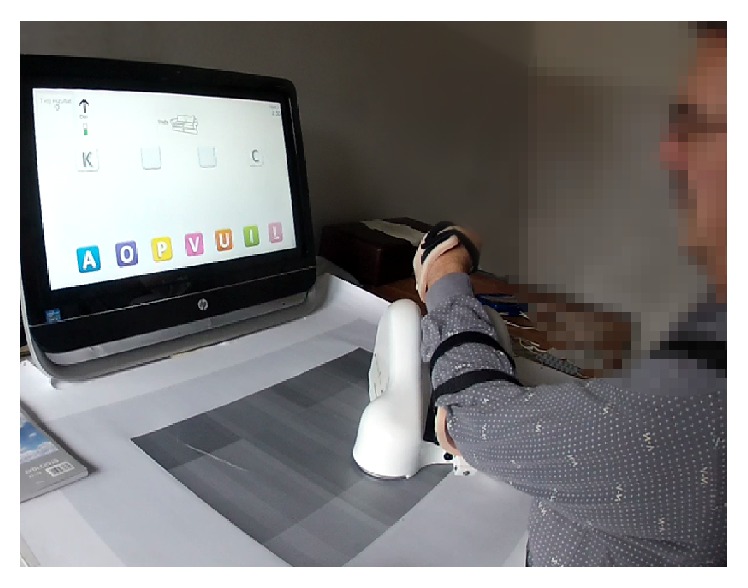
ArmAssist training.

ArmAssist (AA) is a low-cost robotic system developed for shoulder and elbow rehabilitation after stroke (TECNALIA R&I, Spain). It is a modular system that combines a portable device for providing arm support over a table with interactive games operating on a web-based platform (Figure 1). The AA does not actively move the arm; instead, it facilitates active gravity-supported planar arm movements while measuring several parameters (2D position, orientation, forearm angle, and arm support/lifting force) used to control the games. The device is easily attached to the arm by an adjustable forearm and hand orthosis and is designed to allow natural arm movements with minimal resistance. The AA includes games for both training and assessment. The training games include complex tasks that require variable cognitive engagement designed to motivate the user to train longer and more effectively. The examples of training games include puzzles, memory, language, and card games [12]. The assessment games are short tasks (1 to 2 minutes) that evaluate different aspects of the upper limb motor control, such as the range and characteristics of movements with different degrees of freedom. The AA can be used both at a clinic and at home, while the therapist can monitor progress and adapt the therapy accordingly.

The aim of this study was to determine preliminary efficacy of the AA robotic device in comparison to the matched conventional arm training in subacute stroke patients undergoing rehabilitation. The primary outcome measure was Fugl-Meyer Assessment-Upper Extremity (FMA-UE) motor score, and the secondary outcomes were Wolf Motor Function Test-Functional Ability Scale (WMFT-FAS) and Barthel index (BI).

## 2. Methods

### 2.1. Design

We conducted a single-blind (evaluator), two-arm parallel, randomized controlled trial in subacute stroke patients with equal allocation to the two groups. The study was approved by the Ethics Committee of the Clinic for Rehabilitation “Dr. Miroslav Zotović” affiliated with the Faculty of Medicine, University of Belgrade. The study was registered at the ClinicalTrials.gov (NCT02729649) prior to participant enrollment. All participants signed the informed consent form and all research procedures were performed in accordance with the Declaration of Helsinki.

### 2.2. Setting

The study was conducted in a postacute rehabilitation hospital (Clinic for Rehabilitation “Dr. Miroslav Zotović,” Belgrade, Serbia).

### 2.3. Participants

Twenty-six hemiparetic subacute stroke subjects were recruited for this study between January 2015 and March 2016 from the pool of waitlisted patients scheduled for inpatient rehabilitation. During the screening process, all potential participants were interviewed and examined by the study investigator with more than 5 years of clinical experience in neurological rehabilitation. The inclusion criteria were (a) unilateral paresis as a result of first ischemic or hemorrhagic stroke confirmed by computed tomography or MRI that occurred less than 3 months before enrollment, (b) the ability to understand and follow simple instructions, and (c) the ability to perform some active movements in the shoulder and/or elbow joints in the sitting position, allowing for trunk compensation if needed. The exclusion criteria were (a) multiple strokes, (b) bilateral impairment, (c) severe sensory deficits in the paretic upper limb, (d) the inability to provide informed consent, and (e) medical conditions that could interfere with treatment (severe cardiovascular disease, severe visual or auditory impairments, and orthopedic contracture). Baseline FMA in the participants with severe motor impairments was ≤25 and in those with moderate impairments 26 to 50 out of 66 [13]. The participants were randomized using a table of random numbers (SAS software) in the AA group (*n* = 13) or the Control group (*n* = 13).

The sample size calculations were performed using *R* Studio software v. 0.98.976 (Boston, MA, USA), SPSS 17.0 (SPSS Inc., Chicago, IL, USA), and G ^*∗*^Power 3.1. The minimal clinically important difference for FMA-UE was estimated previously at 10% of the maximum score (6.6 points) [14] and adopted here. To achieve at least this difference between the groups and assuming the alpha level of 0.05 and power of 80%, the estimated sample size was 13 per group.

## 3. Interventions

### 3.1. General Overview

The AA group received a conventional rehabilitation with an additional 30 minutes of the AA training. The Control group received the same conventional rehabilitation and an additional 30 minutes of occupational therapy that was matched in its structure and amount to the AA training as close as possible. The AA or matched arm training was administered over 15 sessions each lasting 30 minutes, scheduled 5 days per week (Monday–Friday) for 3 weeks, at least 24 hours apart, and at about the same time of day. Both types of arm training were provided by a physiotherapist with a graduate degree and vast experience in neurological rehabilitation, who was not involved in the assessment. The arm training was carried out in the same therapy room and separated from other treatments by at least a 30-minute break. To assess adverse events, the subjects were asked at the end of each session to report any new symptoms.

### 3.2. Conventional Rehabilitation

The conventional rehabilitation was provided 5 days per week for 3 weeks divided into two 30-minute sessions of occupational therapy and physiotherapy. Occupational therapy for the paretic upper limb included passive stretching within submaximal ranges of motion to inhibit spasticity, active-assisted movements, functional tasks, and activities of daily living, all progressed individually. Physiotherapy consisted of range of motion exercises for upper and lower extremities, gentle stretching, splinting/casting, facilitation of active voluntary movement, and exercises to improve endurance, balance, strength, and gait. If necessary, speech therapy was provided three to five times per week.

### 3.3. ArmAssist Training

The AA training consists of self-directed active movements through interactive gaming with focus on reaching. The movements required for playing the games are horizontal abduction-adduction in the shoulder and flexion-extension in the elbow with the hand in neutral position. The number of reaching movements in each game ranges from 2 to 8 per minute, depending on the cognitive load of the game. In this study, the subjects performed, on average, 4–5 repetitions per minute or about 120–150 movements per session.

The subjects were seated in a chair with a back support and two adjustable shoulder straps to prevent compensatory trunk movements. The straps were adjusted if necessary to allow limited movement of the trunk for comfort. The subject was positioned in the semicircular opening of the table (Figure 1). The chair height was adjusted so the paretic forearm rests on the AA platform with the shoulder in a comfortable position. The AA-based assessment of range of motion and range of force was conducted twice a week (Monday, Friday) to adjust the game difficulty so the subject is challenged but still able to successfully complete the virtual task [12]. The AA robotic system does not have medical certification and has been used in this study as an investigational device.

### 3.4. Conventional Arm Training

**Figure 2:**
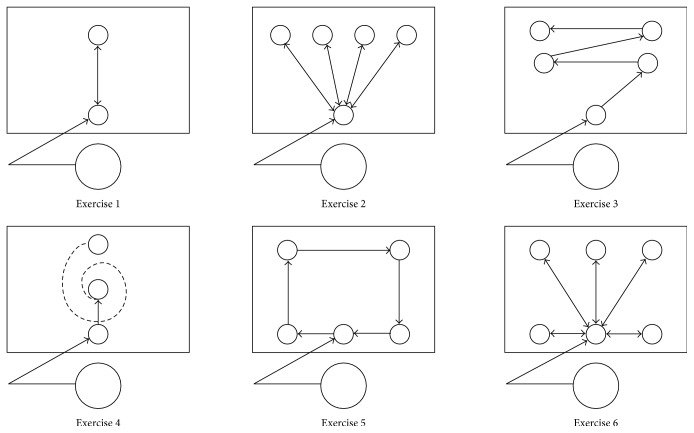
Conventional arm training.

This training consisted of exercises mirroring the structure and amount of AA training as close as possible (Figure 2). The set-up included a chair with adjustable height, a desk, and a cone. The task was to move the cone from the starting position to different target positions (Figure 2), which required horizontal abduction-adduction movements of the shoulder and flexion-extension movements of the elbow, similar to the AA training. The therapist provided assistance as needed and encouraged participants to complete the tasks. The average number of performed movements was estimated at 4-5 per minute or 120–150 repetitions per session, as reported by the therapist and based on the earlier feasibility study conducted in 3 subjects who met the same eligibility criteria.

### 3.5. Outcome Measures

The primary outcome measure was FMA-UE motor score, which assesses the degree of synergistic movements in the paretic upper limb. Individual items pertaining to the shoulder/elbow and hand segments are scored on a 3-point ordinal scale and summed for a maximum possible score of 66 [15, 16].

The secondary outcomes were WMFT and BI. The WMFT is an activity-based test that evaluates upper extremity performance via timed and functional tasks. Each item is rated on a 6-point Functional Ability Scale (FAS) and summed into total WMFT-FAS score. We used a 17-item WMFT, consisting of 15 function-based tasks and two strength tasks, each scored from 0 to 5 for a maximum score of 75 points [17]. The WMFT has shown high reliability and validity for activity-based evaluation of upper limb function [18]. The BI assesses activities of daily living. It includes 10 items which are rated based on the amount of assistance required to complete each activity. The items bathing and grooming are scored 0 or 5; the items feeding, dressing, controlling bladder, controlling bowel, getting on and off the toilet, and ascending and descending stairs are scored 0, 5, or 10. Items regarding transfer from wheelchair to bed and walking on a level surface are scored 0, 5, 10, or 15. The total BI score ranges from 0 (total dependence) to 100 (complete independence) [19]. The BI has proven reliable for assessing rehabilitation outcomes after neurological, neuromuscular, and musculoskeletal diseases [20].

All outcomes were evaluated at baseline and after 3 weeks of intervention by a physiotherapist experienced in neurological rehabilitation, who was blinded to the group allocation (independent evaluator).

### 3.6. Statistical Analysis

Data were analysed using SPSS v17.0 (SPSS Inc., Chicago, IL, USA) and R-studio (2014) v0.98.976 (Boston, MA, USA), following an intention-to-treat analysis using the last forward method. For descriptive purposes, the demographics and baseline outcome measures were compared between the two groups using the independent sample* t*-test (continuous data) and Chi-square or Fisher's test (categorical data). The main outcome measures did not deviate significantly from normality (Kolmogorov-Smirnov test, *p* > 0.05). For the main analysis, the before-after differences were calculated and compared between the two groups using the independent sample* t*-test. The effect size was calculated using Cohen's* d *coefficient (<0.2 small; 0.2–0.8 medium; and >0.8 large) to infer the magnitude of changes. The level of statistical significance was set at *p* < 0.05 (two-tailed).

## 4. Results

**Figure 3:**
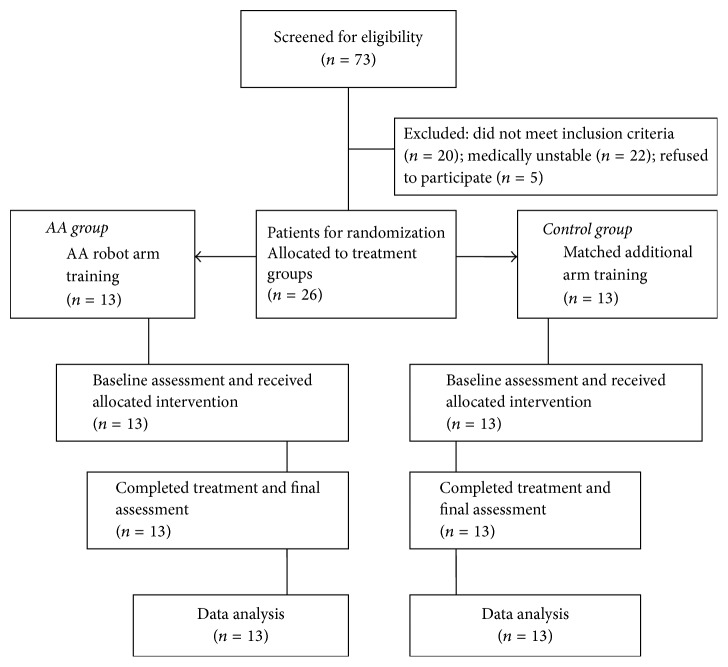
Study flow diagram.

Seventy-three consecutive patients from the waitlist were screened of whom 26 met the eligibility criteria (20 did not meet inclusion criteria, 22 were medically unstable, and 5 refused to participate). None of the 26 recruited subjects dropped out.  Figure 3 shows the flow of subjects through all phases of the study.

There were no significant differences in baseline demographic and clinical parameters between the two groups (Tables 1 and 2). No (serious) adverse events were reported.

The changes in primary and secondary outcomes at the end of treatment are shown in Table 3. There was a significantly greater increase in FMA-UE score (*p* = 0.002) and FMA-UE shoulder/elbow score (*p* = 0.006) in the AA group compared to the Control group. The improvements in WFMT-FAS (*p* = 0.025) and shoulder/elbow portion of WFMT (*p* = 0.010) were also significantly greater in the AA group. All effect sizes were large.

## 5. Discussion

In this study, we compared the efficacy of the AA robotic arm training against the matched conventional arm training in subacute stroke patients with moderate-to-severe arm motor impairment. The results showed that 15 sessions of the AA training resulted in comparably greater reduction in the upper limb motor impairment. The increases in FMA-UE score in both the AA group (18 points) and the conventional group (9 points) were greater than the adopted 6.6-point minimal clinically important difference, suggesting that these gains may be considered meaningful. Moreover, in contrast to most of the previous studies that examined outcomes after robot-assisted arm training [10], our results indicate significant improvements in functional activities (WMFT-FAS score) after using the AA device. These findings could be ascribed to greater reduction in motor impairment, synergistic effects of the AA therapy and spontaneous recovery, or better motivation and greater cognitive engagement while participating in the AA training.

Numerous studies have shown benefits of various robotic devices for improving function of the paretic arm after stroke. Some earlier studies of the robot-assisted arm therapy included chronic and mildly disabled stroke patients [9, 10, 21, 22], whereas more recent studies also showed improvements in subacute patients with moderate-to-severe impairments [23–25]. Recovery of more impaired patients is usually limited and requires intensive and motivating therapy, which the AA device is able to provide.

Our findings are in agreement with the results of previous studies examining effects of low-cost robotic therapy during the subacute phase of recovery from stroke. For example, significant improvements in the upper limb motor performance, as measured by FMA and the Motricity Index, were reported after 2 weeks of robot-assisted treatment in 25 subacute stroke patients [8]. Similarly, a randomized trial of 56 subacute stroke patients with moderate-to-severe disability showed greater increases in FMA and Motricity Index after 15 sessions of robot-assisted training compared to intensive conventional therapy of the same duration [24]. In a single-blind randomized controlled study, Hesse and colleagues found that 30 minutes of robot-assisted arm group therapy combined with 30 minutes of individual arm therapy (5 times per week, 4 weeks) was as effective as a double session of individual arm therapy for reducing the upper limb motor impairment (FMA) in moderately to severely affected subacute stroke patients [25].

In contrast to the FMA-UE and WMFT, we found no significant differences in BI between the two groups. This may be due to a short duration of treatment since BI reflects global physical abilities, which depend on restoration of many other functions and associated comorbidities [26]. No significant difference in BI is consistent with the recent meta-analysis which found evidence of significant improvements in the upper limb motor function after upper limb robotic therapy but no associated changes in the activities of daily living [9].

The improvements after AA training may be due to greater engagement and cognitive demands required for successfully completing virtual gaming tasks. Also, the AA group was required to convert motor action into visual spatial coordinates through interactions with the computer screen. Increased cognitive demands were found to modulate activity in multiple brain motor networks and may enhance motor learning [27].

This study has several limitations consistent with its preliminary nature. We did not consider other factors that could have influenced the outcomes, such as type and location of stroke, motivation, and cognitive abilities. Also, we were able to report an approximate number of repetitions only, whereas the two training protocols were closely matched on several other characteristics of executed movements, such as the type (reaching), plane of action (horizontal), goal (aiming for targets), and constraints (self-directed). No reports of even mild adverse events (e.g., fatigue) with the AA training may reflect an interviewer or response bias. Another limitation is a lack of follow-up to determine durability of effects. Contextual factors such as patient and caregiver impressions should be evaluated in the future. Finally, kinematic analyses are necessary to distinguish improved performance due to arm motor recovery from compensatory movements.

## 6. Conclusions

Arm training using the AA robotic device reduced impairment and activity-related motor deficits more effectively than matched conventional arm training in subacute phase of recovery from stroke. There were no (serious) adverse events. These preliminary results support further research aimed at identifying most suitable candidates and most optimal timing of the AA robotic training as well as feasibility and potential benefits of administration in home settings.

## Figures and Tables

**Table 1:** Demographic data and baseline clinical characteristics.

	AA group	Control group	*p* value^*∗*^
Age (years)^1^	56.5 ± 7.4	58.3 ± 5.2	0.471
Sex^2^			0.322
Male	12 (92.3%)	9 (69.2%)	
Female	1 (7.7%)	4 (30.8%)	
Duration (days)^1^	35.3 ± 9.7	37.3 ± 7.7	0.566
Affected side^2^			0.810
Left	5 (38.5%)	6 (46.2%)	
Right	8 (61.5%)	7 (53.8%)	
Type of stroke^2^			0.617
Ischemic	12 (92.3%)	11 (84.6%)	
Hemorrhagic	1 (7.7%)	2 (15.4%)	
NIHSS^1^	6.1 ± 1.6	6.2 ± 2.2	0.839

NIHSS: National Institute of Health Stroke Scale. ^1^Mean ± standard deviation. ^2^Frequency (percentage). ^*∗*^Statistically significant (*p* < 0.05).

**Table 2:** Baseline outcome measures.

	AA group	Control group	*p* value ^*∗*^
FMA-UE^1^	26.5 ± 7.7	26.6 ± 7.5	0.980
FMA-UE^1^ shoulder/elbow	18.5 ± 6.0	18.7 ± 5.2	0.945
WMFT-FAS^1^	44.2 ± 12.2	42.4 ± 13.3	0.727
WMFT-FAS^1^ shoulder/elbow	24.5 ± 5.5	22.8 ± 4.9	0.419
BI^1^	65.0 ± 26.1	65.4 ± 19.8	0.967

FMA-UE: Fugl-Meyer Assessment of Upper Extremity, WMFT-FAS: Wolf Motor Function Test-Function Ability Scale, BI: Barthel index. ^1^Mean ± standard deviation.  ^*∗*^Statistically significant (*p* < 0.05).

**Table 3:** Changes in outcome measures from the period before to the period after the treatment.

Outcome measure	AA group	Control group	*p*	*d*
FMA-UE^1^	18.0 ± 9.4	7.5 ± 5.5	*0.002* ^*∗*^	*1.909*
FMA-UE^1^ shoulder/elbow	9.1 ± 6.3	3.4 ± 2.6	*0.006* ^*∗*^	*2.192*
WMFT-FAS^1^	14.1 ± 7.9	6.7 ± 7.8	*0.025* ^*∗*^	*0.949*
WMFT-FAS^1^ shoulder/elbow	6.7 ± 7.8	3.2 ± 2.7	*0.010* ^*∗*^	*1.185*
BI^1^	21.2 ± 24.8	13.1 ± 10.7	0.292	—

FMA-UE: Fugl-Meyer Assessment of Upper Extremity. WMFT: Wolf Motor Function Test. BI: Barthel Index. Effect size Cohen's *d* (<0.2 small; 0.2–0.8 medium; and >0.8 large). ^1^Mean ± standard deviation.  ^*∗*^Statistically significant (*p* < 0.05).
